# Assessing the Role of Food Related Lifestyle in Predicting Intention towards Edible Insects

**DOI:** 10.3390/insects11100660

**Published:** 2020-09-25

**Authors:** Fabio Verneau, Francesco La Barbera, Mario Amato, Roberta Riverso, Klaus G. Grunert

**Affiliations:** 1 Department of Political Sciences, Università degli Studi di Napoli Federico II, 80138 Napoli, Italy; verneau@unina.it (F.V.); francesco.labarbera@unina.it (F.L.B.); roberta.riverso@unina.it (R.R.); 2 MAPP Centre, Aarhus University, 8000 Aarhus, Denmark; klg@mgmt.au.dk; 3 School of Marketing and Communication, University of Vaasa, 65101 Vaasa, Finland

**Keywords:** insects, FRLS, perceived behavioural control, intention, consumer behaviour

## Abstract

**Simple Summary:**

The unsustainability of food production is still a major contributor to climate change, therefore utilising new and sustainable food sources is a priority. Edible insects have been part of the human diet for thousands of years but not yet accepted in Western societies, despite a growing literature around the subject. In this paper, we used a holistic approach to understand and predict consumer behaviour in relation to food choices and to edible insects, in particular. Through a questionnaire we collected 300 answers from Italian and Danish consumers; after that, using a statistical model, we divided bystanders into 5 different groups in order to single out consumers who are willing to adopt insects as food. The outcomes of this study have shown that the novelty and benefits of insect consumption have generated much interest in edible insects amongst consumers belonging to the “rational” group, or people who are showing an interested and critical behaviour while shopping for food, who showed the highest intention to eat insects. Therefore, pointing to a group of early adopters, as could be the Rational consumers (20% of our sample), can lay the foundation for a broader commercial development of edible insects with a higher degree of acceptance among consumers.

**Abstract:**

Although recent literature has shown that switching to an insect-based diet could provide several relevant advantages—from a nutritional, environmental, economic and ecological point of view—the potential growth of insects as everyday food is still unclear. Despite a growing literature on consumer acceptance and product preference for insect-based food, a segmentation of this future and possible market has never been proposed. Therefore, in the present paper, a market segmentation based on the Food Related Lifestyle Scale (FRLS), was performed in order to predict consumers’ willingness to eat (WTE) edible insects. Moreover, the role of perceived behavioural control is taken into account. Results shows that the novelty and benefits of insect consumption have generated much interest in edible insects amongst consumers belonging to the Rational cluster, who showed the highest intention to introduce insects in their diet, thus confirming the presence of a niche of “early adopters”. In addition, perceived behavioural control was the major driver of intention. Implications for attempts to encourage people to incorporate insect-based foods into their diet are discussed, with special reference to the role of marketing campaigns.

## 1. Introduction

The unsustainability of food production and consumption has been and continues to be a major contributor to climate change [1,2]. A rampant land grabbing has led to the conversion of natural ecosystems to farmland and pastures, becoming one of the biggest causes of biodiversity loss. If today’s challenges are difficult to face, future humanity will face even greater challenges due to population and food consumption growth [3]. The discovery of new and sustainable approaches for food production, with reduced impacts on atmosphere, land and oceans, is a global priority [4,5].

According to Tukker and Jansen [6], between 20% and 30% of the total human environmental impact is caused by food production. A change of consumers’ lifestyle could reduce environmental impact, for example by replacing animal protein consumption, which is known for having a large environmental impact [7]. As suggested by Premalatha and colleagues [8], replacing beef or pork at least partly by edible insects, which have a comparable protein yield with considerably less feed [9], would be a possible approach. In addition, edible insects possess nutritional advantages in terms of total protein level and/or essential amino acid over plant proteins such as cereals and legumes [10]. The benefits of switching to insect-based foods are not only related to their nutritional value, but also to their positive environmental and economic impact. In fact, compared with conventional livestock farming, farming insects has many advantages including increased feed-conversion efficiency, decreased green-house gases (GHG) emissions, reduced water pollution and smaller land use, with low environmental contamination [11]. Despite these benefits, the growth potential of insects as everyday food is still unclear [12], since widespread consumers’ acceptance of insects as an alternative food source remains a concern [4,13].

Edible insects have been part of the human diet for thousands of years [14], although their consumption is now uncommon in Western societies. There is a growing literature on consumer acceptance and product preference for insect-based food (see [15] for a review). Huge differences in perception, acceptance and willingness to try exist between Eastern countries, where insects have been traditionally used, thereby recognising the nutritional, ecological and economic benefits of entomophagy [16], and Western societies, where a widespread aversion towards consuming insects exists, since this act is not deeply rooted in traditional diet and insects are generally perceived as “unclean”, “mere pests”, “disgusting nuisances” or “disease transmitters” [17,18,19]. Nonetheless, in Western countries and especially in Europe, something is changing. In fact, the recent European regulation 2015/2283 includes insects in the novel food list, making a step forward to a comprehensive and international legal framework [20]. Yet, the process of adoption and implementation into national legislation appears rather slow and fragmented, and this can affect both the availability of insect based products on the market and the perception that consumers have of these novel products [21] Consumer attitudes towards novel food differ noticeably and are guided by factors such as age, gender, knowledge and familiarity, food neophobia, food choice motives, convenience and environmental attitudes [22,23,24]. According to Martins and Pliner [25], consumers’ initial perception of a new food is a crucial factor that affects their willingness to consume. Thus, convincing an insect phobic culture to recognise the relevance of insects for sustainable food supply chains is not only a matter of sustainable production but also of stimulating consumer demand by increasing their acceptance. In the last few years, scholars investigated Westerners’ willingness to accept and adopt insect-based food [26,27,28] or their willingness to substitute meat products with insects [29,30], usually in connection to factors such as food neophobia [18,31,32,33,34,35,36,37], disgust sensitivity [38,39,40,41], previous consumption [31,42,43,44,45,46], indirect measures as implicit associations [47] and other general characteristics such as demographic [28] and general or food-related attitudes [48,49]. Topics such as risk perception, health concern, and social representation of insects as food [50,51,52] are still little explored.

In a more general perspective, from a theoretical point of view, future research should aim to develop a more integrated approach to the study of the antecedents of the willingness to eat insects; on the practical side, studies addressing market segmentation and communication strategies are needed. The Food Related Lifestyle concept (FRL) [53] could be an answer to both those needs, because instead of focusing on single specific factors, the FRL provides a more holistic approach to understand and predict consumer behaviour in relation to food choices. In addition, FRL could be useful for market segmentation and cross-cultural comparisons. To the best of our knowledge, this is the first study utilising FRL in order to predict consumers’ intention to eat edible insects, also providing a market segmentation based on FRL.

Furthermore, even though consumers’ intention to introduce insect-based food into their diet has been widely investigated, at present, an insect-based food market actually does not exist. As mentioned before, since previous research mainly focused on familiarity and/or food neophobia, research should investigate issues related to difficulties that consumers would face if they actually try to introduce this kind of food into their diet (e.g., the unavailability of insect-based food). Therefore, in the current study, this topic was addressed by measuring participants’ perceived behavioural control [54,55] with regard to eating insects, which encompasses their beliefs about the possibility to be autonomous in this choice and the perceived difficulty of the task. In brief, the present paper has two objectives: (1) Segmenting market for insect-based foods using the FRL inventory; (2) analysing the role of perceived behavioural control upon consumers’ intention to eat edible insects.

In the following sections, a brief description of Food Related Lifestyle is provided, followed by an overview of the study and a discussion of the obtained results.

Lifestyle is one of the psychographic criteria that are used in marketing research for market segmentation [56]. As the development of today’s consumer society made socio-demographic characteristics less and less predictive of consumer behaviour, a segmentation using lifestyle was one of the proposals to fill this gap. Scholars suggested the existence of “domain specific lifestyles” [57], i.e., lifestyles that are related to a particular group of products that are linked to a common goal and/or a common purpose. Food is one of the domains that have been studied. The food-related lifestyle concept was developed by Brunsø in 1997 [53,58,59] with the explicit aim to be used as a tool in international segmentation in the food domain. Lifestyle is defined as a cognitive construct encompassing domain-specific declarative and procedural knowledge in the area’s ways of shopping, cooking methods, importance of quality aspects, consumption situations and purchase motives. The FRL model is inspired by the psychological means-end chain theory proposed by Gutman [60] and views lifestyle as a part of a hierarchical, cognitive-behavioural structure which operates as an organisational and guidance construct in a person’s life. Thus, lifestyles are instrumental to achieve individual objectives linked to values (such as hedonism, tradition, self-direction), which are more abstract and trans-situational cognitive categories [61,62,63]. Lifestyles, in certain situations, turn out to be what frames consumers’ perception regarding products and services, guiding her/his choices and behaviours [64]. As noted above, the structure of FRL is expected to consist of five main areas. Two are associated with food purchase motives and food quality aspects, while the other three are connected to food provision, cooking methods and consumption situations. These five cognitive elements are presumed to catch the key characteristics of an individual’s food related lifestyle. The whole model is a system of interacting elements in which personal values are (part of) the underpinning from which purchasing motives are derived; quality aspects, consumption situations, ways of shopping and cooking methods frame our view of food products, services, and other food-related activities and thus affect our behaviour, including food choices and preparation and how we, for example, deal with food and food-related waste [65,66,67,68,69]. The European studies on FRL identified a number of basic cross-cultural food consumer segments that can be found across national borders [59]. These segments are the uninvolved food consumer, the careless food consumer, the conservative food consumer, the rational food consumer and the adventurous food consumer. Analyses have shown that different segments have different food preferences, different perception of food quality and are interested in different types of product information, implying a need for adapting marketing communication towards the specific consumer segments [59]. The instrument has so far been applied in a number of European countries with the purpose of predicting a range of specific food-related behaviours, including how consumers respond to new food products [70], meat consumption [71] and preferences for a vegetarian diet [72].

## 2. Materials and Methods

### 2.1. Data Collection

The study has been conducted in two different European countries, Italy and Denmark, where 300 subjects (150 Danish and 150 Italians) were recruited in the university canteen by a researcher, who approached subjects individually and introduced him/herself as an academic marketing researcher from the local institution (Aarhus University in Denmark, University of Naples Federico II in Italy). After the agreement, participants were led to the lab were computer-based questionnaires were administered. The total procedure took approximately 15 min for each participant to complete. Prior to answer the questions, and in line with Verbeke [49], participants were informed that insects “are a good source of high-value proteins, their production requires little space, their feed conversion is efficient, and therefore the eating of insects provides benefits in terms of sustainability”. The questionnaire contained the FRL inventory, the Intention scale and the Perceived Behavioural Control scale (described below). At the very end, information about gender and level of education were collected. Moreover, participants were also asked about their being vegan/vegetarian and previous consumption experience of insect-based food.

### 2.2. FRL

FRL was measured by the original food-related lifestyle instrument using 69 items (seven-point scales, from “totally disagree” to “totally agree”) to measure 23 lifestyle dimensions, which cover the assessment, preparation and actual consumption of food products, tapping into the five dimension already described: Ways of shopping, quality aspects, cooking methods, consumption situations and purchasing motives [58,59].

### 2.3. Perceived Behavioural Control Scale

Drawing on previous research [54,55], three items were used for measuring perceived behavioural control: (1) I think it is very difficult, for people like me, to introduce insect-based food in their diet (reverse coded); (2) I think that even if I tried, I would not be able to introduce insect-based food into my diet (reverse coded); and (3) In everyday life, each of us could easily introduce insect-based food in her/his diet. Participants answered on 7-point self-anchoring scales from disagree to agree. Items were averaged in a single score (α = 0.81). The higher the score, the higher the perceived behavioural control.

### 2.4. Intention

Three items (adapted from [73]) were used for measuring participants’ intention to introduce insect food in their diet: (1) I would be prepared to eat insect based food in my every day diet; (2) I am willing to buy insect based food if it was available on the market; and (3) I would tell my friends to buy insect based food if it was available on the market. Participants answered on seven-point self-anchoring scales from disagree to agree. Items were averaged in a single score (α = 0.90), therefore higher the score, higher the intention.

### 2.5. Statistical Analysis

Basic descriptive analyses were run on the sample, and measures’ reliability was assessed using Pearson’s alpha coefficient. The FRL dimensions were used to classify participants by hierarchical cluster analysis (Ward’s method). Differences between clusters were tested through ANOVAs. The effect of study variables on consumers’ intention was estimated by means of a linear regression model. Throughout the manuscript, the accepted level of significance for the null hypothesis test is *p* < 0.05. The analysis was performed using SPSS 26 (IBM).

## 3. Results

From the total of 300 recruited subjects, 20 participants were excluded from the analysis; 18 because they declared being vegetarian and/or vegan and 2 because they failed to complete the questionnaire. The final sample consists of 280 subjects (138 females; *M*_age_ = 23.61, *SD*_age_ = 3.86). The two national sub-samples were not significantly different as regards gender (Denmark: 64 females; Italy, 74 females; Χ^2^ (280) = 1.161, *p* = 0.281). Education was dummy-coded (0 = undergraduates, 1 = degree) and was found to be not significantly different in the two subsamples (Denmark: 75 undergraduates; Italy, 85 undergraduates; Χ^2^ (280) = 1.144, *p* = 0.285).

### 3.1. Food-Related Lifestyle

Two subscales were dropped for being not satisfactory as regards reliability (Taste, α = 0.43; Social Event, α = 0.36). The scores of the remaining 21 FRL dimensions were used to classify participants by using hierarchical cluster analysis with Ward’s method. A 5 cluster solution was chosen through the analysis of cluster means, interpretability, and comparability with earlier analyses of FRL data (e.g. [59]). The five clusters emerging were labelled uninvolved, careless, rational, conservative, and adventurous food consumers. Clear differences were observed in the distribution of members of these five groups in the two countries, as can be seen in Table 1.

Almost all participants classified as conservative food consumers were in the Italian sample, whereas most of the uninvolved food consumers were in Denmark. Table 2 provides pairwise comparisons between the means of the 21 dimensions across the five clusters.

According to our data, the five clusters are characterised as follows.

*The uninvolved food consumer*. These consumers are quite uninterested in most aspects of shopping, cooking and eating and have the lowest score of all segments on many of the dimensions. They do attach the highest importance of all segments to convenience, and they have a positive attitude to advertising. These consumers are quite uninterested in most aspects of food, and they hardly use food to achieve their life values.

*The careless food consumer.* The consumers of this segment resemble the uninvolved food segment in many ways, but they have more interest in product information, use specialty shops more often and attach more importance to health and freshness. They also snack more.

*The conservative food consumer.* This segment attaches most importance to attaining security to their food and meal choices and they are most likely to think that cooking is a woman’s task. They put much emphasis on getting fresh products and on getting value for money. They rarely try exotic food recipes and cannot be regarded as novelty-seekers.

*The rational food consumer.* A person belonging to this cluster scores higher on most life-style dimensions than other consumers do, giving rise to an interested while critical shopping behaviour. Product information is especially important to them, and this is important mainly for dietary considerations, moreover they look after prices, use shopping list and enjoy shopping. Regarding cooking methods, they have an above average tendency to look for new ways in the kitchen. Food and food products are an important part of these consumers’ lives and are essential for achieving basic values such as self-fulfilment.

*The adventurous food consumer.* This segment is strongly motivated to try exotic recipes and to buy foods that they have never tried before (they are responsive to novelty). They have a positive attitude to advertising, enjoy shopping and cooking, find fulfilment in cooking and eating and view eating as an occasion to socialise.

### 3.2. Intention

In order to see how intention to adopt insects as part of the diet varies among the five clusters, an ANOVA was performed (Table 3). The effect of the factor FRL clusters on the intention to introduce insect food in the diet was significant, F (4, 275) = 5.001, *p* = 0.001. Post hoc test (method: Bonferroni) showed that the mean scores of rational consumers were significantly higher compared with those of careless and conservative.

In addition, and in line with expectations, a medium significant negative correlation between the scores of intention and perceived behavioural control (r = −0.389, *p* < 0.001) was found. Therefore, the next step was to investigate which variables influenced consumers’ intention, by estimating a linear regression model specified as follows:

*Intention_i_* = *α* + *β_1_* × *Nation_i_* + *β_2_* × *Gender_i_* + *β_3_* × *Education_i_* + *β_4_* × *Perceived Control_i_* + *β_5_* × *Rational_i_* + *β_6_* × *Conservative_i_* + *β_7_* × *Careless_i_* + *β_7_* × *Adventurous_i_* + *ε_i_*

The clusters were dummy coded (keeping uninvolved as the reference category) while perceived control is the average value of the three-item scale. Moreover, Nation, a dummy variable that equals 0 if the subject was Italian, 1 if Danish; Gender, a dummy variable equal to 1 if the subject was female and Education as a dummy variable equal to 1 if the participants possessed a degree, were added as explanatory socio-demographic variables. The model was estimated using STATA 13 software and results are provided in Table 4.

Results shows that several independent variables exerted a statistically significant effect on intention, in particular being Danish rather than Italian, being male and being graduates, as regards socio-demographic characteristics. In addition, perceived behavioural control has a strong positive effect upon intention to consume insect-based foods. Among the FRL clusters, being Rational exerted a positive and statistically significant effect on intention.

## 4. Discussion

The present study has explored, in two different European countries, the potential role of market segmentation using the food-related lifestyle instrument in order to single out consumers who are willing to adopt insects as food. The study is based on Brunsø’s [53] well-established cognitive approach to food related lifestyle segmentation. Five relevant and clearly distinct consumer segments with a meaningful segment size have been defined, in line with previous studies on the FRL, confirming the cross-country validity of the method [74]. In particular, the share of rational food consumers is almost equal in both countries, 19.1% in Italy and 22% in Denmark, and the share of adventurous food consumers is comparable. Main differences can be found in the other three clusters, in fact almost all participants classified as careless and conservative belong to the Italian sample, whereas most of the uninvolved food consumers are Danish. Earlier applications of the FRL in Denmark found conservative food consumers mainly among older consumers, so the dearth of this type in this Danish sample—which consists of young people—is not surprising; finding them in the Italian sample might be related to the conservatism inherent in a strong food culture with considerable heritage and inertia [75,76].

The outcomes of this study have shown that the novelty and benefits of insect consumption have generated much interest in edible insects amongst consumers belonging to the Rational cluster, who showed the highest intention to eat insects. This might be because Rational consumers have critical and interested shopping behaviours. According to the results, they look after prices and actually enjoy shopping in order to satisfy their need to find new cooking methods or recipe. Moreover, they have a strong interest in healthy products and novelties, and they are more willing to gather information while shopping compared to other clusters. Therefore, this higher level of intention might partly be explained by the information note that consumers received at the beginning of the study, in which insects’ properties were described, probably stimulating the interest of Rational consumers. Of course, a link between reported intention to consume edible insect and actual future consumption cannot be stated, even though “early adopters”, as rational consumers can be defined, merit attention and further research.

Importantly, perceived behavioural control emerged as a strong prediction of the intention to eat insects. In the current study, PBC regards individual beliefs about the ease or difficulty of obtaining or consuming insect food. Irrespectively to the intention to consume edible insects, people might actually feel it is impossible to perform the behaviour, because of edible insects’ low availability on the market and little/no knowledge of the product and how it can be utilised. A demand for new foodstuffs is affected by increases in supply [76,77,78,79], therefore “a particular food must be widely available if it is to become an accepted and integrated part of people’s diet” [80]. Therefore, taking into account that perceived behavioural control can affect behaviour indirectly by its impact on intention, major marketing strategies should focus on the positive and distinctive attributes of edible insects, both from a nutritional and an environmental perspective, but also on their availability on the market, on individuals’ knowledge of different insect-based products, and of ways to cook them [9]. Considering the fact that new foods gain popularity in one small segment of society first, before diffusing further, as it has been the case with tea [77] or sushi [81], pointing to a group of early adopters, as could be the Rational consumers (20% of our sample), can lay the foundation for a broader commercial development with a higher degree of acceptance among consumers.

This research has several limitations. First and foremost, despite studies in the literature demonstrating that student samples do not intrinsically pose a problem for a study’s external validity [82], a broader and more stratified sample is required in further research. In addition, in this work the role of disgust in general was not explored and, more importantly, disgust towards insects as food because of the lack of an instrument that directly addresses this issue in literature. The absence of disgust among predictive factors could be among the reasons why the proportion of variance explained by the statistical model employed was relatively small. This research, therefore, should foster new studies in other European Countries, maybe analysing the different perception of edible insects between the East and West also in the light of the different legislative approaches adopted in different countries that may affect the degree of consumer awareness; replicating the use of Food Related Lifestyle and perceived behavioural control, also exploring the connection between these variables and the disgust, which has been the more evoked antecedent of Westerners’ aversion towards entomophagy, still needs investigation.

## 5. Conclusions

The present work is one of the first attempts to perform a market segmentation using, as a main key, the Food Related Lifestyle Scale in order to predict consumers’ willingness to eat edible insects. The use of the FRL inventory highlighted the main differences between Italian and Danish sample. Moreover, the intention to introduce insects into everyday diet is higher in the Rational cluster of consumers, that can be considered as a niche of early adopters of the products. Finally, the perceived behavioural control has been found as a major driver of intention, opening new avenues for addressing marketing campaigns.

## Figures and Tables

**Table 1:** Distribution of Food Related Lifestyle concept (FRL) clusters over countries.

	Uninvolved	Careless	Conservative	Rational	Adventurous	Total
Italy	10.6%	19.1%	29.8%	19.1%	21.3%	100%
Denmark	39.7%	5.7%	1.4%	22.0%	31.2%	100%
Total	25.2%	12.4%	15.6%	20.6%	26.2%	

**Table 2:** Distribution of FRL clusters over countries.

	Uninvolved	Careless	Conservative	Rational	Adventurous
**Product info**	4.55 ^a^	5.56 ^b,c^	6.06 ^b,c^	6.21 ^c^	5.48 ^b^
**Advertise attitude**	4.01 ^b^	3.23 ^a^	3.25 ^a^	2.96 ^a^	4.00 ^b^
**Enjoy shopping**	4.22 ^a^	4.28 ^a^	5.07 ^b^	5.61 ^b^	5.18 ^b^
**Speciality shops**	3.89 ^a^	4.74 ^b^	5.53 ^c^	5.60 ^c^	4.19 ^a^
**Price criteria**	5.15	5.30	5.47	5.65	5.47
**Shop list**	4.36 ^a^	5.12 ^a,b^	5.55 ^b,c^	6.04 ^c^	4.59 ^a^
**Health**	3.46 ^a^	5.59 ^b^	5.51 ^b^	5.85 ^b^	3.99 ^a^
**Price quality**	4.55 ^a^	5.72 ^b,c^	6.14 ^c^	6.13 ^c^	5.37 ^b^
**Novelty**	4.33 ^a^	4.16 ^a^	3.80 ^a^	5.51 ^b^	5.18 ^b^
**Organic**	3.13 ^a^	5.34 ^b^	4.93 ^b^	5.51 ^b^	3.28 ^a^
**Freshness**	4.43 ^a^	6.29 ^c^	6.45 ^c^	6.00 ^c^	5.22 ^b^
**Cooking int.**	4.32 ^b^	3.33 ^a^	5.95 ^c^	6.16 ^c^	5.84 ^c^
**New way**	3.96 ^a^	4.13 ^a,b^	4.80 ^b^	6.16 ^c^	5.66 ^c^
**Convenience**	2.87 ^c^	2.51 ^b,c^	1.64 ^a^	1.75 ^a^	2.17 ^a,b^
**Whole family**	3.74 ^a^	3.50 ^a^	4.80 ^b,c^	5.24 ^c^	4.30 ^a,b^
**Planning**	3.91 ^b^	3.15 ^a^	3.28 ^a,b^	3.67 ^a,b^	3.47 ^a,b^
**Woman task**	2.26 ^a^	3.16 ^b^	3.35 ^b^	1.55 ^a^	1.88 ^a^
**Snack meal**	2.90 ^a,b^	4.30 ^c^	3.66 ^b,c^	2.52 ^a^	3.51 ^b,c^
**Fulfilment**	4.07 ^a^	4.12 ^a^	5.27 ^b^	5.84 ^b^	5.26 ^b^
**Security**	3.06 ^a^	4.19 ^b^	4.36 ^b^	2.61 ^a^	3.00 ^a^

Note: In each line, different letters mean equal mean—Bonferroni method.

**Table 3:** Mean scores of intention across clusters.

FRL Clusters	*M*	*SD*	*N*
**Uninvolved**	4.00 ^a,b^	1.58	70
**Careless**	3.39 ^a^	1.86	35
**Conservative**	3.26 ^a^	1.91	44
**Rational**	4.70 ^b^	1.92	57
**Adventurous**	4.01 ^a,b^	1.78	74
**Total**	3.95	1.84	280

Note: In each line, different letters mean equal mean—Bonferroni method.

**Table 4:** Regression model of the Intention to eat insects.

Model	Coefficient	*t*	*p*
**Nation**	−0.178	−2.976	0.003
**Gender**	0.122	2.286	0.023
**Education**	0.159	3.058	0.002
**Perceived control**	0.379	7.213	0.000
**Rational**	0.172	2.655	0.008
**Conservative**	−0.060	−0.862	0.390
**Careless**	−0.035	−0.560	0.576
**Adventurous**	0.018	0.283	0.777
**(Constant)**		14.603	0.000

Note: Dependent variable: Intention. R^2^ = 0.28, *F* (1, 271) = 13.16, *p* < 0.001.
